# Factors that determine depth perception of trapezoids, windsurfers, runways

**DOI:** 10.3389/fnhum.2015.00182

**Published:** 2015-04-09

**Authors:** Chia-Huei Tseng, Joetta L. Gobell, George Sperling

**Affiliations:** ^1^Department of Psychology, The University of Hong KongHong Kong, China; ^2^Frank N. Magid, Associates, Inc., New YorkNY, USA; ^3^Department of Cognitive Sciences and Department of Neurobiology and Behavior, Institute of Mathematical Behavioral Sciences, University of California, Irvine, IrvineCA, USA

**Keywords:** depth perception, ambiguous scenes, frame of reference, trapezoid illusion, windsurfer illusion, runway illusion

## Abstract

We report here a windsurfer^1^ illusion, a naturally occurring trapezoidal illusion in which the small end of the sail viewed at a distance appears to be pointed away from the observer even when it is closer. This naturally occurring illusion is so compelling that observers are unaware of their gross perceptual misinterpretation of the scene. Four laboratory experiment of this kind of trapezoidal illusion investigated the joint effects of retinal orientation, head position, relative motion, and the relative direction of gravity on automatic depth perception. Observers viewed two adjacent white trapezoids outlined on a black background rotating back and forth ± 20° on a vertical axis much like the sails of two adjacent windsurfers. Observers reported which side of the trapezoids (long or short) appeared to be closer to them (i.e., in front). The longer edge of the trapezoid was reported in front 76 ± 2% of trials (“windsurfer effect”) whether it was on the left or on the right. When the display was rotated 90°to produce a runway configuration, there was a striking asymmetry: the long edge was perceived to be in front 97% when it was on the bottom but only 43% when it was on top (“runway effect”). The runway effect persisted when the head was tilted 90° or when displays on the ceiling were viewed from the floor. Ninety-five percent of the variance of the variance in the strikingly different 3D perceptions produced by the same 2D trapezoid image was quantitatively explained by a model that assumes there are just three additive bias factors that account for perceiving an edge as closer: Implicit linear perspective, lower position on the retina (based on an automatic assumption of viewing from above), and being lower in world coordinates.

## Introduction

There often are situations in which the information available to the visual system is consistent with multiple interpretations. In most cases, the visual system chooses one of these interpretations to resolve the ambiguity, usually without the viewer being conscious of the ambiguity. When the visual system’s conclusion does not accord with the physical reality of the scene, an illusory perception occurs. In this paper, several related trapezoidal illusions pertaining to the perceived 3D configuration of windsurfers and of airplane runways are displayed and investigated.

### The Windsurfer Illusion

**FIGURE 1:**
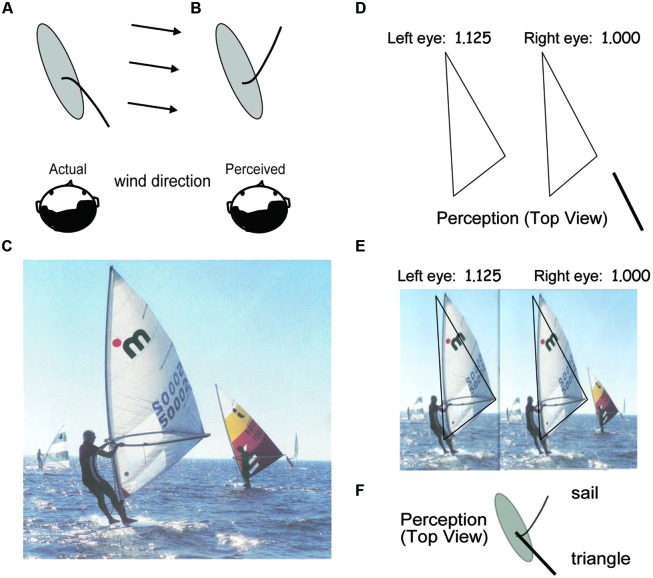
**Example of the windsurfer illusion. (A)** The veridical and **(B)** the illusory top view of a windsurfer traveling up and to the left. The gray ellipse represents the windsurfer board on which the sailor stands, the curved line represents a sail cross-section, and the head represents a distant viewer. **(C)** A photograph of the scene. The veridical perception **(A)** is difficult to achieve. Instead, the sail appears concave to the left as in **(B)** despite clear information to the contrary (e.g., if the sail were actually in the illusory position, the sailor would be unsupported and immediately fall into the water). **(D)** Left and right half-images of a stereogram triangle, the left image is expanded 1.125 horizontally. When viewed stereoscopically, the right vertex of the sail appears much nearer than the vertical edge (as illustrated below). **(E)** Left- and right-half images of the triangle superimposed on a left-expanded windsurfer image providing a strong stereo cue to indicate that the vertex opposite the vertical side is nearer the viewer than is the vertical side. **(F)** Top view of a windsurfer board (gray ellipse), the sail, and the triangle.

The illusion that initially motivated this study occurs when viewing a windsurfer or group of windsurfers from a distance. The end of the sail consistently appears to be pointed away from the viewer, even when the position of the surfer, the direction of the wind, and all other physical aspects of the situation would make such an arrangement impossible. **Figure 1C** contains a photograph illustrating the illusion.

The middle corner of the sail in **Figure 1C** appears to be pointed away from the observer. For most viewers, this illusion is so compelling and so difficult to reverse that the diagrams of **Figures 1A,B** are necessary to explain that what is perceived actually is an illusion, a completely incorrect interpretation of the depth orientation of the sail. To illustrate how persistent this Windsurfer illusion is, even in the presence of stereo depth cues, a triangle in approximately the same shape as the sail is illustrated in **Figure 1D**.

**Figure 1D** contains two halves of a stereogram of a triangle in which the left eye’s image has been expanded by 1.125 in the horizontal dimension only. This stereogram is designed to be viewed from a distance of about 20 cm. When the two half-images are binocularly fused, they produce a strong stereo-depth effect in which the right vertex of the triangle appears to be much closer to the viewer than the vertical edge (as illustrated below). In **Figure 1E**, the image of the windsurfer is similarly expanded horizontally to provide a similar depth cue to assist the viewer in perceiving the vertex of the windsurfer sail as being closer to the viewer than the vertical edge. The same triangle as in **Figure 1D** is superimposed to outline the sail. A common stereo perception in **Figure 1D** is that the triangle’s vertex opposite the vertical side appears closer to the observer than the vertical side as in **Figure 1F**, but the windsurfer illusion stubbornly persists. This illustrates that it is not merely the gross outline shape of the sail, but other cues that make veridical perception difficult. One such cue is the lighting in the photograph which makes the far side of the sail (which is near the mast) much lighter than the near side which is darker. The experiments reported below deal only with the shape component of the Windsurfer illusion, so much remains still to be explored.

One way to reverse the windsurfer illusion of **Figure 1C** is to stare at the picture for an extended period, on the order of many minutes, until it “flips,” i.e., is perceived in its correct orientation (**Figure 1B**). Difficult as that perception is to achieve, once achieved it is the obviously correct one.

### Context Influences the 3D Interpretations of 2D Shapes

**FIGURE 2:**
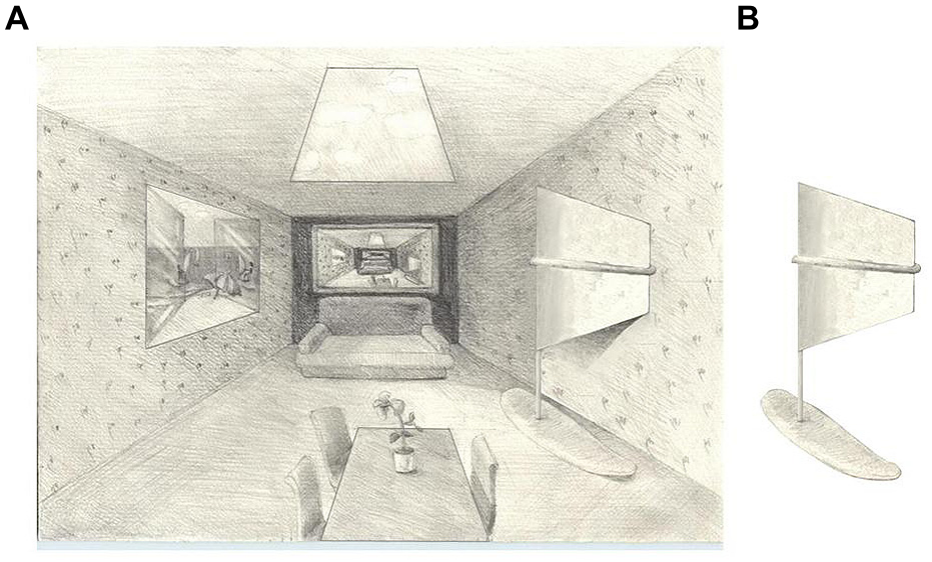
**Context can determine the 3D interpretations of 2D shapes. (A)** The table and skylight are identical in 2D shape, yet their perceived shapes are quite different due in part to the 3D context of the scene. The 2D trapezoids on the left and right wall also are identical shapes but they represent apparently different 3D shapes. On the other hand, compare the depth orientations of the window on the left wall, the windsurfer sail on the right wall of **(A)** and the isolated windsurfer sail in **(B)**.

The windsurfer illusion is an example of an illusion that depends primarily on the 2D image of a 3D object, i.e., it is fairly free of context that might influence the 3D interpretation. More generally, however, the perceived 3D shape of the same physical 2D shape can be heavily influenced by context. **Figure 2** illustrates how the appearance of the same 2D trapezoidal outline (the subject matter of the present experiments) depends in a logical way on the pictorial context in which it is placed, the walls of a 2D representation of a 3D cubicle.

All four outline shapes in **Figure 2A**, the table, skylight, window, and windsurfer sail, are identical except for a translation or rotation. The scene context determines the perceived 3D shape of these objects, but has less effect on the perceived depth. In **Figure 2A**, although both the table and the window on the left wall are recognized as trapezoids, they are simultaneously recognized as representing 3D rectangular objects whereas the skylight and the windsurfer sail are recognized as non-rectangular. On the other hand, the window on the left wall, the windsurfer in the room, and the isolated windsurfer in **Figure 2B** all appear to have generally similar depth orientations.

### Frame of Reference Determines the Perception of “Above”

In addition to the importance of pictorial context, studies have shown that the determination of the direction “up” has a strong influence on how certain ambiguous stimuli are interpreted. For example, in the absence of information about illumination, observers tend perceive convexity and concavity in accordance with the implicit perceptual assumption that the illumination is coming from above (Yonas et al., 1979; Howard et al., 1990; Jenkin et al., 2004). However, the interpretation of “above” can have difference reference points, e.g., the head or retina (here, “retinal” coordinates), and gravity (here, “world” coordinates; e.g., Rock, 1973). Rock suggested that environmental vertical was what one usually aligned with object presentation, and when objects were presented in unfamiliar orientation, we tended to assume that shapes were upright in the environment (Rock and Heimer, 1957; Rock and Nijhawan, 1989). In fact, when reporting their percepts of convex and concave surfaces, Yonas et al. (1979) reported that 4-years-old children use their head positions more than gravity as the determination of “up.” Older children (7-years-olds), on the other hand, seem to use head position, and gravity fairly equally.

Howard et al. (1990) investigated the influence of retinal and world frames of reference in adult observers. Their data indicated that the assumption about the direction of illumination–and by inference, “above”–was predominantly determined by retinal configuration, and that world coordinates were used only when the retinal configuration was uninformative, and even then world coordinates were used somewhat inconsistently. Jenkin et al. (2004) also manipulated frames of reference in a task in which adult observers adjusted the orientation of shaded disks such that they appeared maximally convex or concave. They determined that observers’ responses varied systematically with changes in frames of reference, indicating that both retinal and world coordinates play a role in the perception of “above.”

### Rotating Ames Trapezoid

Ames (1951) constructed a trapezoidal shaped frame that rotated 360° around a vertical axis. He noted that such a trapezoid is perceived as repeatedly reversing its rotational direction, even though no such reversals occur. In natural viewing, a major component of this reversal is the trapezoidal illusion, the tendency to see the long side of the trapezoid as nearer than the far side, and this would require perceptual depth-and-direction-of-rotation reversals every 180° of rotation. Following Ames’s initial observation, many researchers investigated the factors that increased or decreased the number and/or frequency of apparent reversals. Factors investigated include perspective (Zenhausern, 1969; Martinetti, 1975; Braden, 1978), motion parallax (Graham, 1963; Braden, 1978), luminance (Canestrari and Farne, 1969), shadowing and interposition (Cross and Cross, 1969), interior texture (Day and Power, 1965; Cahill, 1975; Braunstein and Stern, 1980), incomplete versus complete figure boundaries (Cook and Mefferd, 1966; Braunstein and Stern, 1980; Mitchell and Power, 1983), speed of rotation (Braden, 1978) and apparent orientation (Day and Power, 1965).

The Ames-related studies enumerated above required observers to indicate any motion reversals perceived during continuous rotation of the trapezoidal figure. The factor of interest was then manipulated to determine if it had a significant effect on the number or frequency of reversals reported. The manipulations of perspective, motion parallax, luminance, shadowing, interposition, and apparent orientation had such an effect, while interior texture and figure boundary completion did not.

#### Fatigue

A trapezoid rotating through 360° consists of a sequence of images; the images change in a systematic way and at a rate determined by the experimenter. This image trajectory involves several complex processes including fatigue and object momentum. Fatigue refers to the tendency of ambiguous figures to produce an alternation between the possible perceptions, presumably because the neurons generating the active percept become “fatigued” permitting the alternative percept to become active. For instance, Long et al. (1992) reported that the longer observers viewed an unambiguous version of a Necker cube, the more likely an ambiguous version of the cube was perceived in the opposite configuration.

#### Object Momentum

Object momentum refers to the tendency, in the short term, to continue to see the same object through changing views. In the motion domain, this concept has been noted in the positive distortion (of memory or perception) of an object’s final position along a movement path, referred to as representational momentum (Freyd, 1987). This effect has been documented in vertical and horizontal translational motion (Hubbard and Bharucha, 1988) and, more relevant to our investigation, in rotational motion as well (Munger et al., 1999). Munger et al. (1999) found distortions of the reported final rotational position of a rotating tilted cube such that observers seemed to see the cube stop further along the rotational path than its actual stopping point. Such representational momentum in rotation has implications for reported depth perception whenever a rotating trapezoid crosses the frontoparallel plane. At that point, whether an edge that has been coming forward in depth continues to come forward or reverses its depth direction depends on the rotational representational momentum because the objective depth is momentarily completely ambiguous. Thus, an Ames paradigm is, in effect, a series of successive trials that occur every 180° of rotation when the rotating trapezoid is exactly in the fronto-parallel plane. At this point, representational momentum (continuing in a circular direction) is put into competition with the many other factors that determine direction reversal.

#### Outline of the Present Study

Although our goal, ultimately, is to understand the windsurfer trapezoidal illusion, given current technology, the windsurfer situation is too complex too study. The Ames trapezoid is an idealized simplification and has the advantage of having already generated extensive useful research. However, even the rotating Ames window is unnecessarily complicated as it involves viewing trapezoids at all possible angles, and an implicit succession of consecutive trials as the trapezoid repeatedly passes through the front-parallel plane. To further simplify and focus the study of trapezoidal illusions, we consider only one typical orientation of an Ames-like trapezoid. The dependent variable is which side of the trapezoid appears nearer the observer. The perception of depth in a trapezoid is greatly enhanced, as in the Ames window, by rotating it, in this case, rotating the trapezoid back-and-forth around its original orientation. Such rotation typically produces a vivid percept of depth the moment the trapezoid is perceived. A third factor is the control of fixation point. Our solution is to show two rotating trapezoids with the observer visually fixated at a fixation point in between them.

Given a 2D trapezoidal stimulus that is nearly always perceived as rotating in 3D and a simple dependent variable (which side is closer), the following factors were studied individually and in combination: (1) location of the axis of rotation, (2) orientation of the trapezoids (windsurfer or runway), (3) various combinations of retinal orientation and world orientation (gravity). A model is proposed that accounts very efficiently for the resulting data.

#### Visual Displays

Although a static 2D trapezoid is usually perceived to 3D depth, once a trapezoid is set into rotary motion, the depth perception becomes stronger and typically is bistable–only one of two configurations is perceived, the observer can easily indicate which one by reporting whether the long trapezoidal edge was closer or further away than the short edge, and the observer is unaware of the alternative depth configuration. In the bistable 3D perception of a rotating trapezoid, the perception of 3D depth is instantaneous and automatic. That is, the instant the display is perceived, it is perceived as having 3D depth. This automatic process of 3D depth perception of trapezoids is the object of the present study.

**FIGURE 3:**
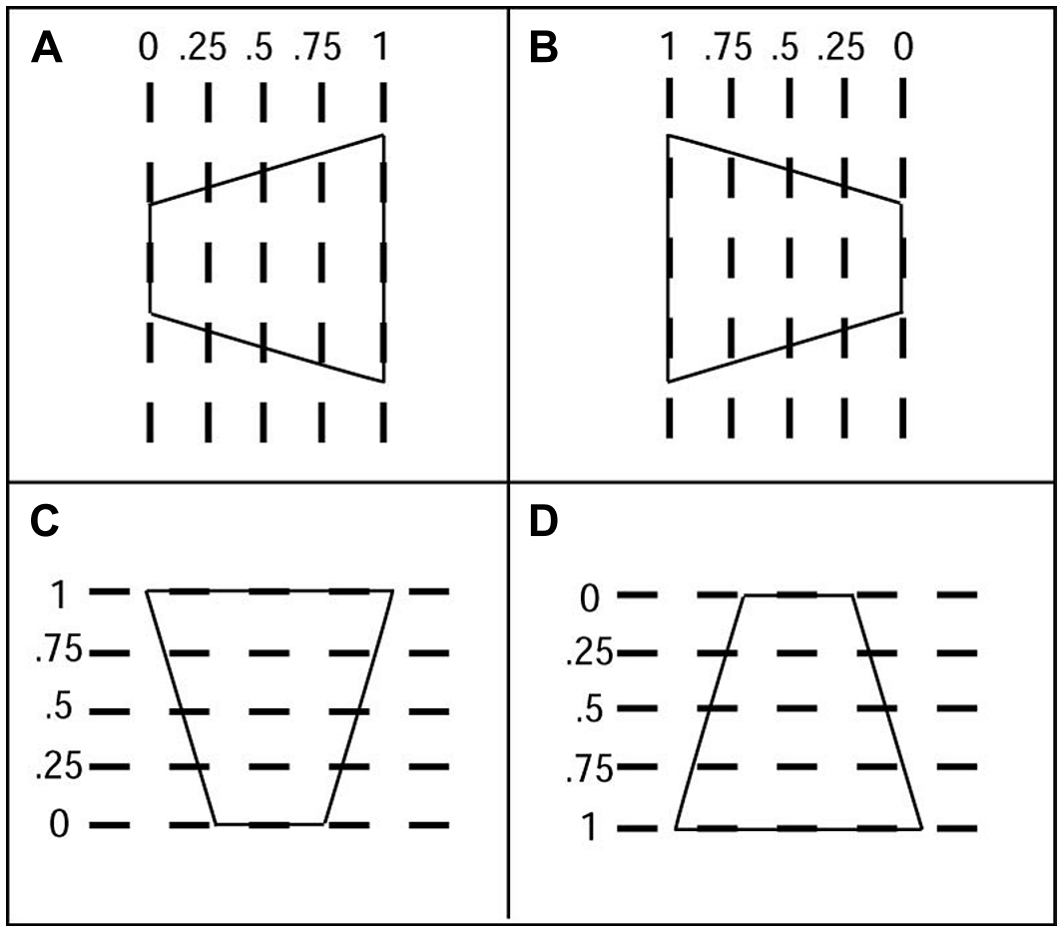
**Axes-of-rotation for four display configurations. (A)** Display with the windsurfer orientation, short-side-left. **(B)** Windsurfer orientation, short-side-right. **(C)** Runway orientation, short-side-down. **(D)** Runway orientation, short-side-up. Displayed movements were two 20-deg back-and-forth 3D rotations in depth about one of five vertical axes dashed lines in **(A,B)** and about horizontal axes **(C,D)**. All five axis orientations were tested. The 3D rotation speed was 70°deg/s. The trapezoids were depicted under parallel projection, which simulates infinite viewing distance.

The stimulus orientations in **Figures 3A,B** are similar to the situation in which a windsurfer’s sail rotates about its mast when in use, and is therefore termed the windsurfer orientation. The orientations in **Figures 3C,D** are similar to the situation in which a ground or ceiling plane extends away from the observer, and is therefore termed the runway orientation.

## General Method

Observers viewed trapezoidal outlines that rotated back and forth (oscillated) about a rotational axis (See **Figure 3**) and indicated with a button press which side appeared closer. The oscillations were restricted to ± 20° rather than undergoing full 360° rotations around a vertical axis as in classical Ames studies. As noted above, the classical Ames (1951) stimulus varies enormously as the angle changes, and we wished to determine a perceptual response that was not averaged over many different configurations but rather determined by just small variations around a typical configuration and in a brief time interval. The narrow range of oscillation minimizes the effects of representational momentum on depth perception. The exposure duration was sufficiently brief (2.2 s) that reversals (perceptual alternations) rarely occurred. We propose to study only the initial perceptual state that is produced “automatically” by a particular stimulus orientation and not how this state evolves during an extended observation period. All methods used were approved by the UC Irvine Institutional Review Board, and all participants provided signed informed consent forms.

## Experiment 1: Windsurfer Illusion

Experiment 1 determines how the position of the axis of rotation influences the observer’s perception of the oscillating trapezoids in the windsurfer orientation.

Motion parallax predicts that the side of the trapezoid which exhibits “more” motion (a combination of the velocity and of the length of moving edge) would be seen in front because movement close to the observer produces greater velocities and larger retinal images than that same movement further away from the observer. So, when one edge is moving much more quickly than the other, the visual system should perceive it as closer than the side with less motion. Guastella (1966) suggested with reference to trapezoids rotating through 360°, it may be that the “observer attends to the physical parameter which is changing at the greater rate” (also Graham, 1963).

### Stimuli

Observers were presented displays containing trapezoidal outlines in which the location of the axis of rotation for a particular trial was chosen randomly from among five equally spaced locations, ranging from coincident with the short side to coincident with the long side. **Figures 3A,B** illustrate the five possible locations for the axis of rotation.

Stimuli were displayed using a computer-driven CRT, at a resolution of 480 × 640 pixels, and a refresh rate of 120 Hz. Stimuli were generated in MATLAB, using the Psychophysics Toolbox extensions (Brainard, 1997; Pelli, 1997). The stimulus display consisted of white outlines of two trapezoidal shapes and a central white fixation point on a black background (0.2 cd/m^2^). The stimuli were viewed in a dark room. Trapezoid lines were 8.75 × 10^-5^ cd/m for horizontal lines and 8.58 × 10^-5^ cd/m for vertical lines.

**FIGURE 4:**
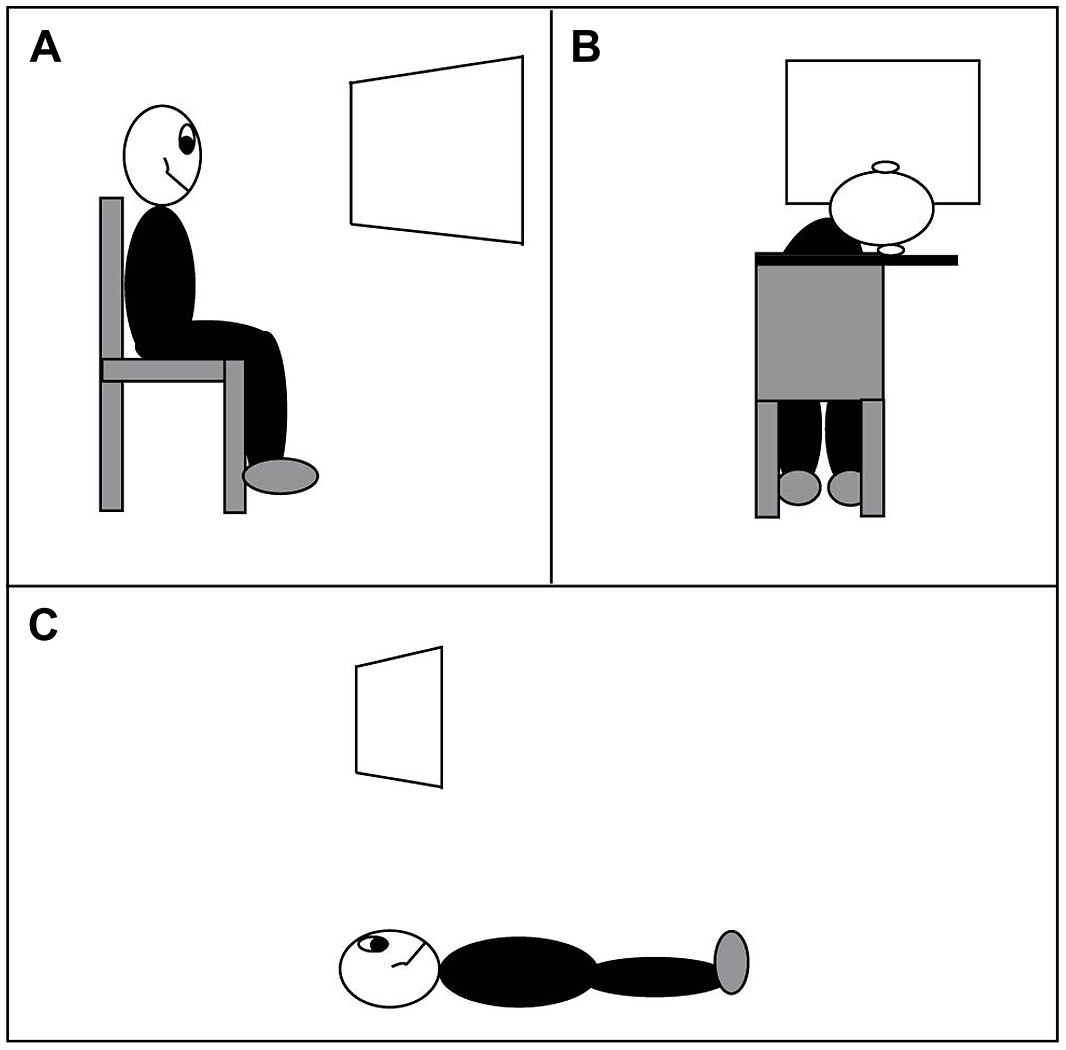
**Three viewing conditions. (A)** Normal viewing, head vertical. **(B)** Head rotated 90° to the right. **(C)** Lying on the floor, viewing a display on the ceiling.

The long side of the trapezoid subtended 4.43° of visual angle, the short side subtended 2.22° of visual angle, and the maximum distance between the two parallel sides of a trapezoid was 4.43° of visual angle. The lengths of long and short sides did not change during a rotation (parallel projection) and they remained constant throughout the experiments. The distance from the center of one trapezoid to the center of the other was 8.86° of visual angle. The viewing distance was 90 cm, and observers viewed the display monocularly with their preferred eye while sitting upright (the “vertical viewing” condition, **Figure 4A**).

**FIGURE 5:**
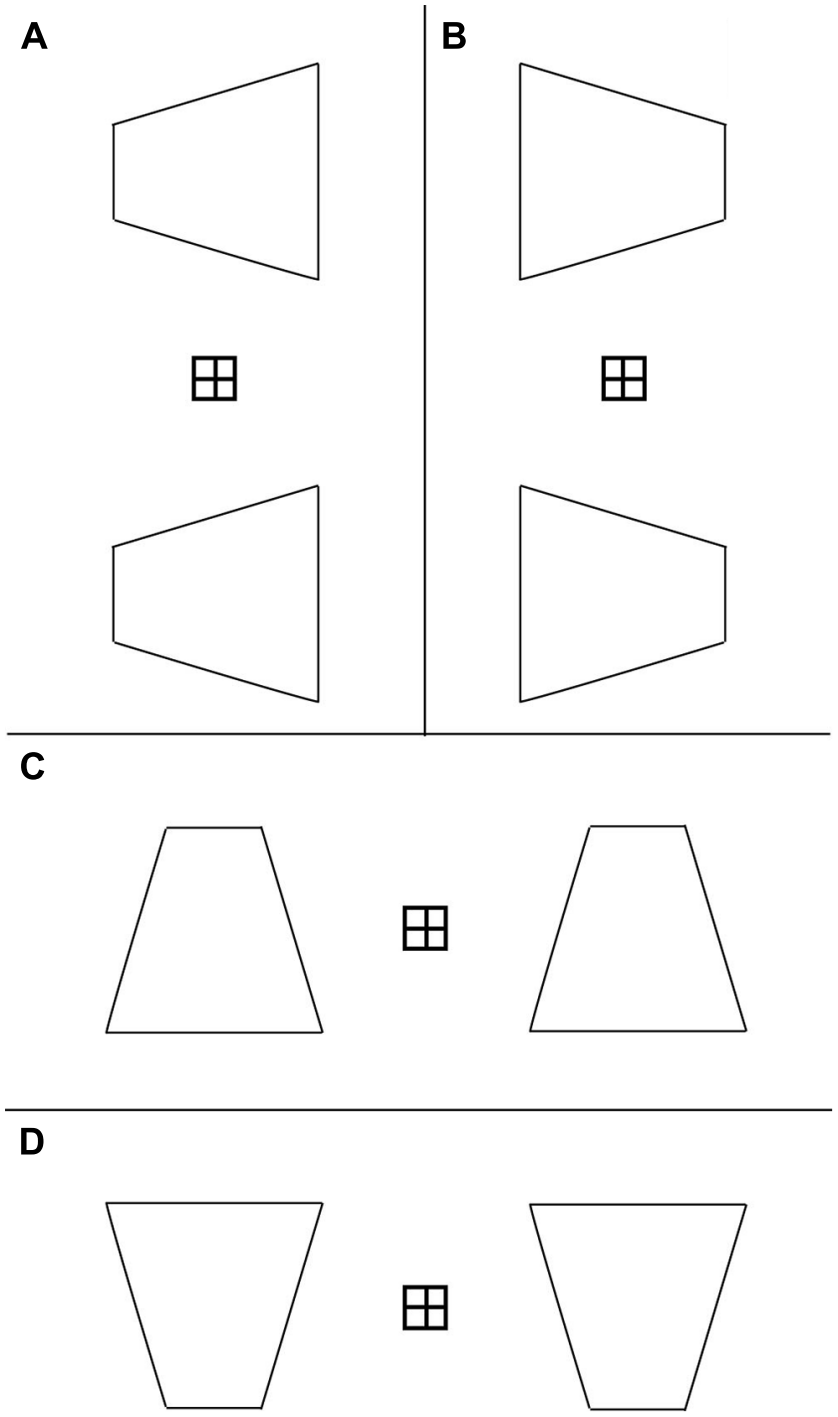
**Four stimulus configurations used in current experiments: **(A)** windsurfer, short side left, **(B)** windsurfer, short side right, **(C)** runway, short side top, **(D)** runway, short side bottom**.

The windsurfer display consists of two trapezoids, one located above and one below the fixation point, oriented with their parallel sides vertical (**Figures 5A,B**). The left–right orientation of the trapezoids was chosen randomly on each trial – the short side appeared on either the left or the right side of the display (i.e., on both trapezoids) with equal probability. Thus, the two possible stimulus orientations were:

Stim = (Ret, Short*__l_*; Wrd, Short*__l_*), and Stim = (Ret, Short*__r_*; Wrd, Short*__r_*), where Ret indicates retinal configuration, Wrd indicates world configuration (gravity-based), and subscripts *l* or *r* indicate that the short side of the trapezoids was to the left or right.

The reason for displaying two trapezoids in each display was to allow the fixation point to be centered with regard to the long and short sides of the figures without the addition of any interior elements inside the trapezoids.

### Procedure

Observers viewed stimuli of moving outline trapezoids on a computer screen. Observers were seated upright in a chair in front of the CRT, henceforth referred to as the vertical viewing condition. Viewing was monocular with the preferred eye; the other eye was covered with an eye patch. On each trial, the trapezoids oscillated (rotated in depth and then reversed the direction of rotation) twice through ± 20° about one of five randomly chosen axes of rotation. Each stimulus display lasted 2.32 s (two back-and-forth oscillations). At the completion of the rotating trapezoid display, a blank screen appeared until the observer registered his or her response, and the next trial began immediately.

All judgments were of the nature: “Which side is closer to you in depth?” and were indicated by key presses. Possible responses were as follows:

(1) short side in front throughout entire trial,
(2) long side in front throughout entire trial,
(3) short side in front at beginning of trial, changing at least once during the trial,
(4) long side in front at beginning of trial, changing at least once during the trial
(5) out of phase–the two trapezoids are in opposite depth configurations, and
(6) not a three dimensional percept.

When the observer responded that the trapezoids were moving out of phase (5), s/he was then required to report the depth configuration of each trapezoid separately (1–4). These categories were developed based on numerous preliminary experiments and accounted for essentially all the observers’ perceptions. A related observation throughout these experiments is that, when an observer is perceiving a particular perceptual mode (i.e., the short side in front), the observer usually is not aware of the possibility of other modes.

### Subjects

Seven observers completed two blocks of 200 trials each, for a total of 400 trials. Each condition has 40 observations times 7 observers.

### Results

#### Conditional Proportions

Although observers were provided with the six possible responses outlined in the previous section, 96% of the total responses were “short side in front throughout entire trial” or “long side in front throughout entire trial.” It will be convenient, therefore, in describing the data and in subsequent discussion to divide the responses into two groups: Group 1: short-in-front and long-in-front; Group 2: all other responses, i.e., response categories 3–6. Subsequently, when we write proportion of short-in-front responses or long-in-front responses, we refer to the proportion within Group 1 so that the proportions of short-in-front plus long-in-front responses add to 1.0, and either proportion can be easily inferred from the other. When referring to the proportions of responses in categories 3–6, we use unconditional (absolute) proportions.

#### Trapezoidal Illusion

**FIGURE 6:**
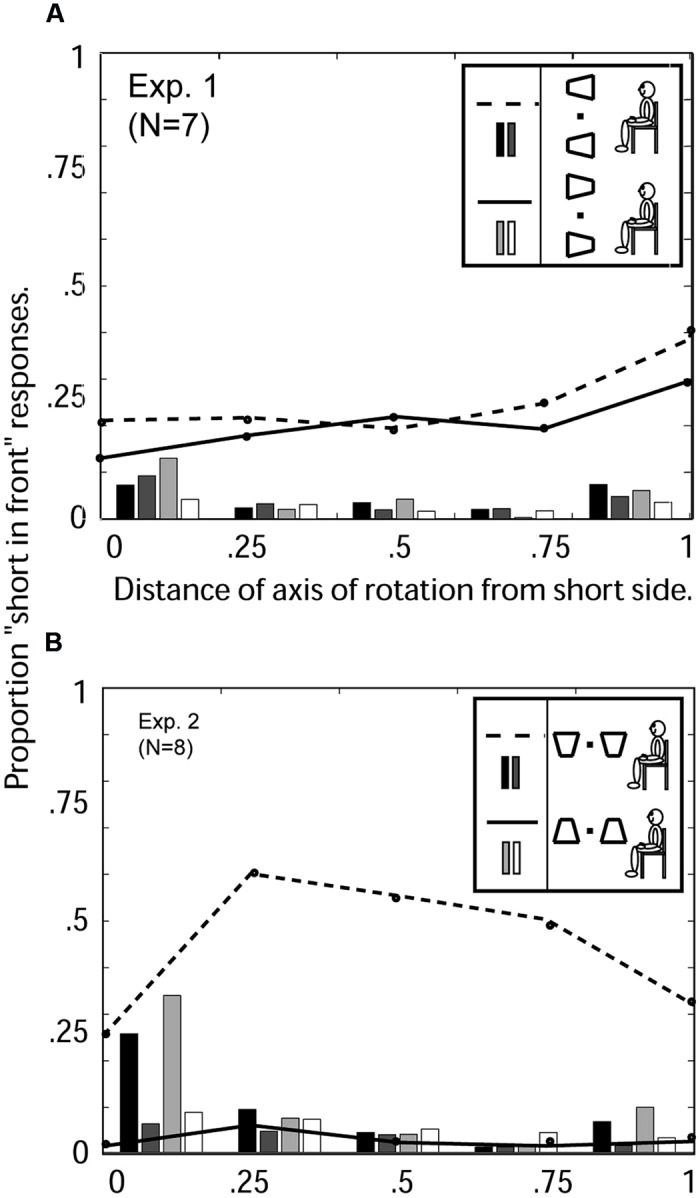
**Experiments 1 and 2: Average 3D perceptions of seven observers of oscillating windsurfer trapezoids and runway trapezoids viewed with the head vertical as a function of the location of the axis of rotation. (A)** Experiment 1: Windsurfer world orientation and windsurfer retinal orientation. Abscissa: 0 indicates the axis of rotation was coincident with the short trapezoid side; 1 indicates the axis was coincident with the long side (see **Figure 3**). For the orientation with short side displayed on the left (see insert), the three response categories are indicated as follows: the dashed line indicates “short-side-in-front” responses, the black bars indicate the proportion of trials on which no depth was perceived, and dark gray bars indicate the proportion of trials in which the two simultaneous trapezoids were perceived in opposite depth configurations. For the orientation with short side displayed on the right, the corresponding response categories are indicated by solid line, light gray bars, and white bars (see insert). **(B)** Experiment 2: Runway world orientation, runway retinal orientation. Dashed lines, and black and dark gray bars, are from trials in which the short side of the trapezoid was displayed on the bottom. Solid lines, and light gray and white bars, are from trials in which the short side of the trapezoid was displayed on top (see insert).

**Figure 6** shows the percent of “short-in-front” responses for each of the five axis of rotation positions (0, 0.25, 0.5, 0.75, 1), beginning with the axis at the short side (0), and continuing to the axis at the long side (1). The first and most obvious result is that the proportion of short-in-front responses varies between 15 and 39%, with an average of 26%. Alternatively stated, the conditional probability of seeing the long side in front (versus the short side) is 74%. The strong tendency to perceive the long versus the short side in front reflects a strong trapezoidal illusion at all locations of the axis of rotation.

#### The Effect of Location of Axis of Rotation on Perceptual Organization

The hypothesized effect of axis of rotation is insignificant. The hypothesized effect of motion strength – an increase in probability of the short side being perceived in front the more it moved – would produce a positive slope in the data of **Figure 6A**. Indeed, the data appear to show a positive slope but, by an Analysis of Variance, the effect of “axis-of-rotation” is not statistically significant [*F*(13,56) = 1.29, *p* < 0.24].

#### Left–Right Asymmetry?

Another question of interest is whether the pattern of responses was similar for the two possible orientations of the trapezoids (short side on the left or short side on the right). These two conditions are represented by dashed line and solid line respectively. The pattern of responses was statistically identical for both orientations. In the windsurfer configuration, left–right asymmetry is negligible and statistically completely insignificant.

#### Other Response Categories

The absolute proportion of response other than left- or right-in-front for the entire trial is 4%. There is not much we can say about these relatively infrequent responses. In **Figure 6A**, they combine into two categories: (i) no apparent motion in depth and (ii) more than one perception during the 2 s observation interval. The main observation here –and it will be more obvious later—is that when the axis of rotation is at the short side, there is a higher proportion of trials in which the trapezoid appears to remain flat in the frontoparallel plane—i.e., a change-in-shape versus motion-in-depth is perceived.

#### Individual Differences

There are clear and obvious general patterns in the data averaged over observers but there also are substantial individual differences. The range of individual differences is consistent with findings from earlier studies utilizing the Ames window rotating through 360° (e.g., Mulholland, 1956). Individual differences were found in the overall proportion of short-in-front responses, in the effect of axis of rotation on depth perception, and in the interaction of these two factors. The model we consider below to account for the group data could be expanded to account for individual differences. However, the amount of data required to establish parameters for each individual is an order of magnitude greater than what is available from these experiments, so we necessarily concentrate here on factors that are common to the population of observers.

## Experiment 2: Runway Illusion

Because the determination of “up” and “down” in a scene is important to its subsequent interpretation (e.g., Jenkin et al., 2004), here we investigate the effect of a global 90° rotation of the windsurfer display about the fixation point. Such a rotation changes “up” and “down” in the scene, and produces what we refer to as the runway orientation (see **Figure 5**).

Based on research suggesting a bias for observers to interpret scenes as though viewing from above (Mamassian and Landy, 1998) and research indicating a bias to see the lower portion of the visual field as closer in depth (Dunn et al., 1965), it was hypothesized that observers would be most likely to report the side which was at the bottom of the figure as appearing closest in depth.

The main depth-determining cues in Experiment 1 were implicit trapezoidal perspective and amount of motion. In Experiment 2, the displays are identical except for a global rotation of the entire display 90 counterclockwise. Therefore, the same factors as in Experiment 1 are again relevant: implicit perspective (except that now the effect of perspective depends on whether the display is perceived as viewed from above or below, versus previously, left or right), and the amount of motion (the same five axes-of-rotation locations are again investigated).

### Methods

#### Stimuli

All aspects of the stimuli were identical to Experiment 1 except that the entire display was rotated 90° counterclockwise (see **Figure 6** inserts). The two possible stimulus configurations are: Stim = (Ret, Short*__t_*; Wrd, Short*__t_*), and Stim = (Ret, Short*__b_*; Wrd, Short*__b_*), where Ret indicates retinal configuration, Wrd indicates world configuration (gravity-based), and *t* and *b* indicate that the short side of the trapezoids was at the top or bottom, respectively.

#### Procedure

The procedure in Experiment 2 is identical to that in Experiment 1.

#### Subjects

The seven of the observers of Experiment 1 plus one new observer participated in Experiment 2.

### Results

#### Trapezoidal Illusion Overruled

**FIGURE 7:**
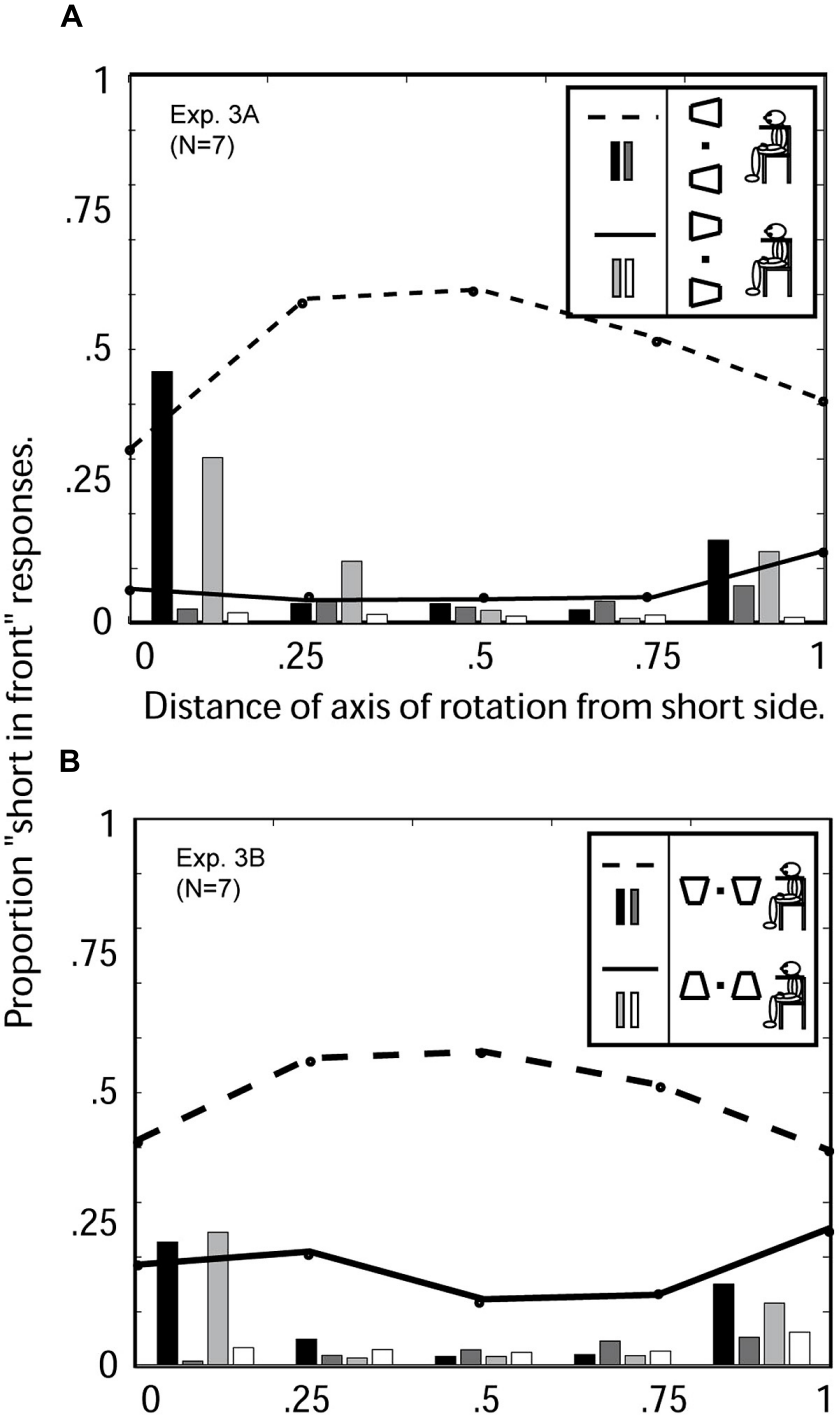
**Experiment 3: average 3D perceptions of seven observers of oscillating windsurfer and runway trapezoids as a function of the location of the axis of trapezoid rotation when viewed with the head tilted 90° to the right. (A)** Experiment 3a: Windsurfer world orientation, runway retinal orientation. Abscissa: 0 indicates the axis of rotation was coincident with the short trapezoid side; 1 indicates the axis was coincident with the long side. For the orientation with short side displayed on the left (see insert) the three response categories are indicated as follows: the dashed line indicates “short-side-in-front” responses, the dark gray bars indicate the proportion of trials on which the two simultaneous trapezoids were perceived in opposite depth configurations, the black bars indicate the proportion of trials in which no depth was perceived. For the orientation with short side displayed on the right, the corresponding response categories are indicated by solid line, white bars, and light gray bars (see insert). **(B)** Experiment 3b: runway world orientation, windsurfer retinal orientation. Dashed lines, and black and dark gray bars, are from trials in which the short side of the trapezoid was displayed on the bottom. Solid lines, and light gray and white bars, are from trials in which the short side of the trapezoid was displayed on top (see insert). Left–right symmetry is broken (solid and broken data curves are separated) when either retinal position or gravity indicate an up–down component to the display.

**Figure 6B** shows the percentage of “short in front” responses for each axis of rotation location for the two runway world configurations (short side on top and short side on bottom). There is a clear and systematic pattern in the responses; namely, when the short side of the trapezoid is toward the top of the display, it is virtually never perceived to be in front. When the short side is on the bottom of the display it is perceived to be in front 57% of the time. Fifty-seven percent short-in-front (i) is significantly greater than in the windsurfer configuration (Experiment 1, **Figure 7A**) when the short side was perceived in front only 26% of the time (averaged over observers and axis positions), and (ii) represents a very significant asymmetry between the two configurations (left and right before rotation, now up and down). The response symmetry between left- and right- pointing trapezoids in Experiment 1 is completely broken by the enormous response asymmetry between up- and down-pointing trapezoids. The up–down asymmetry is consistent across all observers.

According to informal observer reports, when the long side is toward the bottom of the display, the runway is perceived as being approximately rectangular in shape and viewed in perspective. This creates the clear impression of a floor surface. When the short side is toward the bottom of the display, the runway is perceived as trapezoidal in shape. If the trapezoid were perceived as approximately rectangular in shape and viewed in perspective, this would be consistent with a surface on the ceiling viewed from below. The tendency to see the bottom side in front is consistent with the hypothesis that the runway display tends to be interpreted as being viewed from above regardless of which orientation is being viewed. We refer to the bias to see the bottom side of the rotating trapezoid as forward in depth as the “bottom-side-in-front bias” or the “runway effect.”

#### Other response Categories

As in Experiment 1, when the axis of rotation was coincident with the short side, the experience of a failure of depth occurred more frequently than in other axis locations. In Experiment 2, the frequency of failures to perceive depth in the condition with the axis of rotation coincident with the short side varied from 10–55% of trials for individual observers with a mean frequency of 26% (versus 11% in Experiment 1) In other words, instead of appearing to be an outline shape oscillating about a rotational axis, on 26% of trials, overall, the stimulus appeared to be a frontoparallel trapezoid distorting in shape.

### Discussion

The data from the windsurfer configuration of Experiment 1, averaged over observers and positions of the axis of rotation, indicate that the long side of the trapezoid is perceived in front on 76% of the trials. We can regard 76% as indicating the perceptual effectiveness of implicit perspective. In Experiment 2, when the long side is on the bottom, it is perceived in front on 97% of the trials (implicit perspective and bottom-side-in-front are in agreement). When the long side is displayed on top, it is perceived in front only 43% of the time (implicit perspective and bottom-side-in-front bias are in conflict). If perspective and bottom-side-in-front bias were of equal strength, we would expect 50% long-side-in-front judgments. That 43% is observed suggests that bottom-side-in-front actually overrules implicit perspective.

With no change in the stimulus other than a 90° rotation, a consistent and significant change in perceived depth and shape occurs for all observers. We refer the pattern of results, the strong tendency to perceive the bottom-side versus the top side as “in front” as the *runway effect*.

## Experiment 3: World vs. Retinal Configuration

Experiment 3 was conducted to determine the respective contributions from the world and retinal configuration. In Experiment 2, the scene was rotated 90° about the fixation point relative to Experiment 1 while the viewing position remained unchanged. Rotation of image meant that the coordinates of the stimulus were changing in terms of coordinates outside of the observer (world configuration) as well as in terms of the coordinates on the observer’s retina (retinal configuration). The radical and consistent change in depth percept observed in Experiment 2 could, therefore, be due to the change in either or both coordinate systems. In order to decorrelate the contributions of retinal and world coordinates, observers viewed both windsurfer and runway world configurations with their heads tilted at a 90° angle, resting on a cushioned support. Together with Experiments 1 and 2, the data will provide the two different retinal displays each viewed in two different world positions (relative to gravity), i.e., all combinations of retinal and world position for the displays.

### Method

#### Stimuli

All aspects of the displayed stimuli themselves in Experiment 3 were identical to Experiments 1 and 2. Both the windsurfer and runway world configuration were used. The four possible stimulus configurations were:

Stim = (Ret, Short*__l_*; Wrd, Short*__b_*), Stim = (Ret, Short*__r_*; Wrd, Short*__t_*), Stim = (Ret, Short*__t_*; Wrd, Short*__l_*), and Stim = (Ret, Short*__b_*; Wrd, Short*__r_*), where Ret indicates retinal configuration, Wrd indicates world configuration (gravity-based), and *l, r, t* and *b* indicate that the short side of the trapezoids was to the left, right, top, and bottom, respectively.

#### Procedure

In Experiment 3, the observers completed the same task as in Experiments 1 (Experiment 3A, windsurfer display) and 2 (Experiment 3B, runway display) with their heads tilted 90° to the right, referred to as the horizontal viewing condition (**Figure 4B**).

### Results

The results from Experiment 3 are summarized in **Figure 7**. A response asymmetry typical of a runway configuration as in Experiment 2 (**Figure 6B**) was preserved in both conditions in current Experiment (**Figures 7A,B**), indicting the runway perceptual up–down asymmetry overrides the left–right symmetry of the windsurfer configuration. The runway effect (bottom-side-in-front bias) is somewhat greater in retinal coordinates than world coordinates.

In order to compare the effects of a change in retinal configuration and a change in world configuration, cross-comparisons with data from Experiments 1 and 2 are required. The comparison of Experiments 1 and 3a, or of Experiments 2 and 3b, allows the investigation of what happens when the retinal configuration changes and the world configuration remains the same. The comparison of Experiments 1 and 3b, or of Experiments 2 and 3a, allows the investigation of what happens when the world configuration changes and the retinal configuration remains the same.

#### Changing Retinal Configuration

##### Windsurfer world configuration [Experiment 1 (**Figure 6A**) versus Experiment 3A (**Figure 7A**)]

For all observers, changing the retinal configuration from windsurfer to runway concurrent with a world windsurfer configuration produces a profound change in the pattern of responses, changing almost perfect left- versus right-pointing trapezoid response symmetry into the highly asymmetrical bottom-side-in-front response bias. However, the implicit perspective interpretation is maintained so that all observers are significantly more likely to report the long side in front than the short side.

##### Runway world configuration [Experiment 2 (**Figure 6B**) versus Experiment 3B (**Figure 7B**)]

The runway effect that was observed in Experiment 2 remained strong in Experiment 3B. However, tilting the head 90° in Experiment 3B, changes the retinal runway into a retinal windsurfer configuration, and thereby reduces overall bottom-side-in-front bias (runway effect) by about 10%. This shows that a retinal configuration consistent with the world configuration (Experiment 1) contributes to the ultimate perception—both retinal and world configurations are important.

#### Changing the World Configuration

##### Windsurfer retinal configuration [Experiment 1 (**Figure 6A**) versus Experiment 3B (**Figure 7B**)]

The retinal windsurfer configuration remains constant while world configurations changes from windsurfer to runway. The change in world configuration from windsurfer to runway generates a strong bottom-side-in-front bias (runway effect).

##### Runway retinal configuration [Experiment 2 (**Figure 6B**) versus Exp 3A (**Figure 7A**)]

For all observers, leaving the retinal configuration in the runway configuration while changing the world configuration from runway to windsurfer produces very little change in the overall pattern of responses. This is not to say that there are not any changes for some observers, but rather that the runway effect present in the data from Experiment 2 remains present in Experiment 3A for all observers and in the same amount in the average data. The runway effect in retinal runway configuration is so strong that a world windsurfer configuration (relative to a world runway configuration) insignificantly weakens the runway effect. Both world are retinal runway effects are powerful, but the retinal effect is stronger.

### Discussion

Our data show that neither the retinal nor world coordinates exclusively determine perception for any of the observers. To our knowledge, there is no existing model for evaluating the strength of these coordinate frames and to show how information from separate coordinates is combined for perceptual decision for stimuli like the Ames rotating window or the rotating trapezoid in our displays. In the last section, we offer a model to quantitatively describe the component influences on trapezoid perception.

## Experiment 4: Viewing From Below

In Experiment 2, it was hypothesized and confirmed that the short side of the trapezoid would be reported closer in depth with greater frequency when it appeared on the bottom of the runway orientation than when it appeared on the top. The high consistency of responses across observers suggests the consistent involvement of similar higher-level visual processes in the resolution of the depth ambiguity. However, it was not clear from Experiment 3 what assumptions and/or calculations about the visual environment and what information from other senses about the direction of gravity the visual system may have applied in resolving these perceptual ambiguities.

Experiment 4 further investigates the possibility that the runway effect is the result of a bias to perceive objects as if viewing from above (Mamassian and Landy, 1998). This experiment addresses what happens to the depth orientation of the trapezoids when observers were presented with very strong cues signaling viewing from below. Observers viewed the runway orientation projected on the ceiling while lying on the floor looking upward (**Figure 4C**). With such clear cues provided by gravity and the visual orientation of the environment, the observer is placed in a situation highly inconsistent with viewing from above. Therefore, the reclining posture should weaken a bias to interpret a scene as being viewed from above. If the runway effect persists in this viewing condition, it would suggest that the runway bias was induced by cues or factors other than “viewing from above.”

### Stimuli

While the general stimulus configuration remained similar to Experiment 2, there were several differences as follows: The stimuli were displayed using a computer-driven CRT, with a resolution of 480 × 640 pixels, and a refresh rate of 120 Hz, then projected using a Proxima Desktop Projector 4200 and a front-silvered mirror positioned in front of the projector at a 45°angle. Stimuli thereby projected onto a smooth white ceiling.

In Experiment 4, only the runway orientation was displayed because preliminary observations indicated that there was perfect symmetry in viewing short-side left and short-side right windsurfer configurations. The two possible runway configurations are: Stim = (Ret, Short*__t_*, and Stim = (Ret, Short*__r_*), where Ret indicates retinal configuration, and* t* and *b* indicate that the short side of the trapezoids was to the top (head), and bottom (feet), respectively, of the observer. The world configuration did not correspond to either the short side at the top or bottom. The viewing distance was approximately 210 cm. The long side of the trapezoid subtended 3.1° of visual angle, the short side subtended 1.55° of visual angle, and the distance between the two parallel sides of a trapezoid was 3.1° of visual angle. The distance from the center of one trapezoid to the center of the other was 6.2° of visual angle. Overall, the display size in terms of visual angle was slightly smaller (0.70) than the size of the displays in Experiments 1–3.

### Procedure

Six observers from among those participating in the Experiments 1–3 viewed stimuli of moving outline trapezoids projected onto a white surface on the ceiling. Observers were positioned below the projection screen, lying on their backs, so that the screen was frontoparallel. The observers were positioned such that the fixation point was directly vertical above the nose. All other aspects of the experiment, including response options, were identical to those in Experiments 1–3.

Under the “viewing-from-above” hypothesis, it would be expected that the runway effect would be diminished in this experiment because the observer is viewing the runway orientation in a manner that is inconsistent with a bias for viewing from above according to (1) gravity and (2) the visual environment. Howard et al. (1990) construe “above” as referring (3) to the long axis of the body (i.e., “head-centered above”) or with respect to (4) the retinal projection of the visual field (“retinal above”). The ceiling viewing procedure eliminates “viewing from above” in senses (1) and (2) but not (3) and (4) which were dealt with in Experiments 1–3.

### Results

**FIGURE 8:**
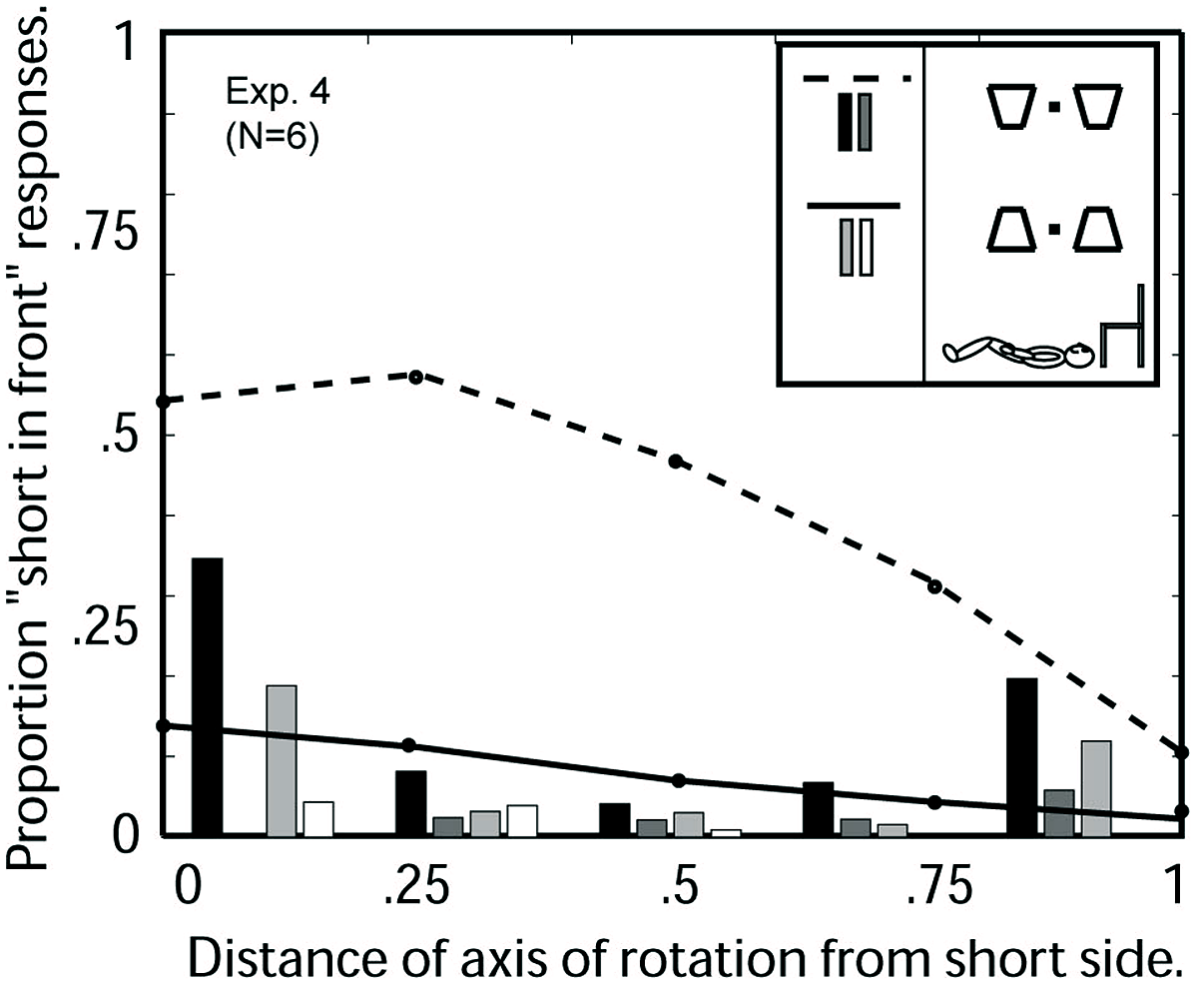
**Experiment 4: Average 3D perceptions of six observers viewing oscillating trapezoids in a runway configuration on the ceiling while lying on the floor as a function of the location of the axis of rotation.** Abscissa: 0 indicates the axis of rotation was coincident with the short trapezoid side; 1 indicates the axis was coincident with the long side. For the orientation with short side displayed on the bottom (see insert), the three response categories are indicated as follows: the dashed line indicates “short-side-in-front” responses, the black bars indicate the proportion of trials on which the two simultaneous trapezoids were perceived in opposite depth configurations, the gray bars indicate the proportion of trials in which no depth was perceived. For the configuration with short side displayed on the top, the corresponding response categories are indicated by solid line, light gray bars, white bars.

**Figure 8** shows the results of Experiment 4. There is a strong runway effect (bottom-side-in-front bias) although the runway effect is somewhat reduced as compared to observers viewing the same images sitting upright (Experiment 2, **Figure 6B**).

## A Quantitative Model for Evaluating and Combining Factors that Bias Depth Perception in Rotating Trapezoids

There are many possible comparisons between the four experiments for evaluating the factors that control the perception of depth. Rather than consider these comparisons individually, we propose a quantitative model to account for the effects of three factors in all the experiments.

### The Data to be Accounted for

The four experiments described here involved ten different stimulus conditions in which observers judged the apparent depth configuration of rotating trapezoids that oscillated back and forth. Here we use the proportion of long-in-front responses (1 – short-in-front) for convenience because it yields more positive versus negative numbers. The model considers only the proportion of long-in-front responses relative to the short-in-front responses, and does not consider the less frequent responses such as shape change without 3D depth and different depth orientations for the two displayed trapezoids. This subset of the results is averaged over observers and over the five different axes of rotation yielding one datum for each of the 10 viewing conditions.

**FIGURE 9:**
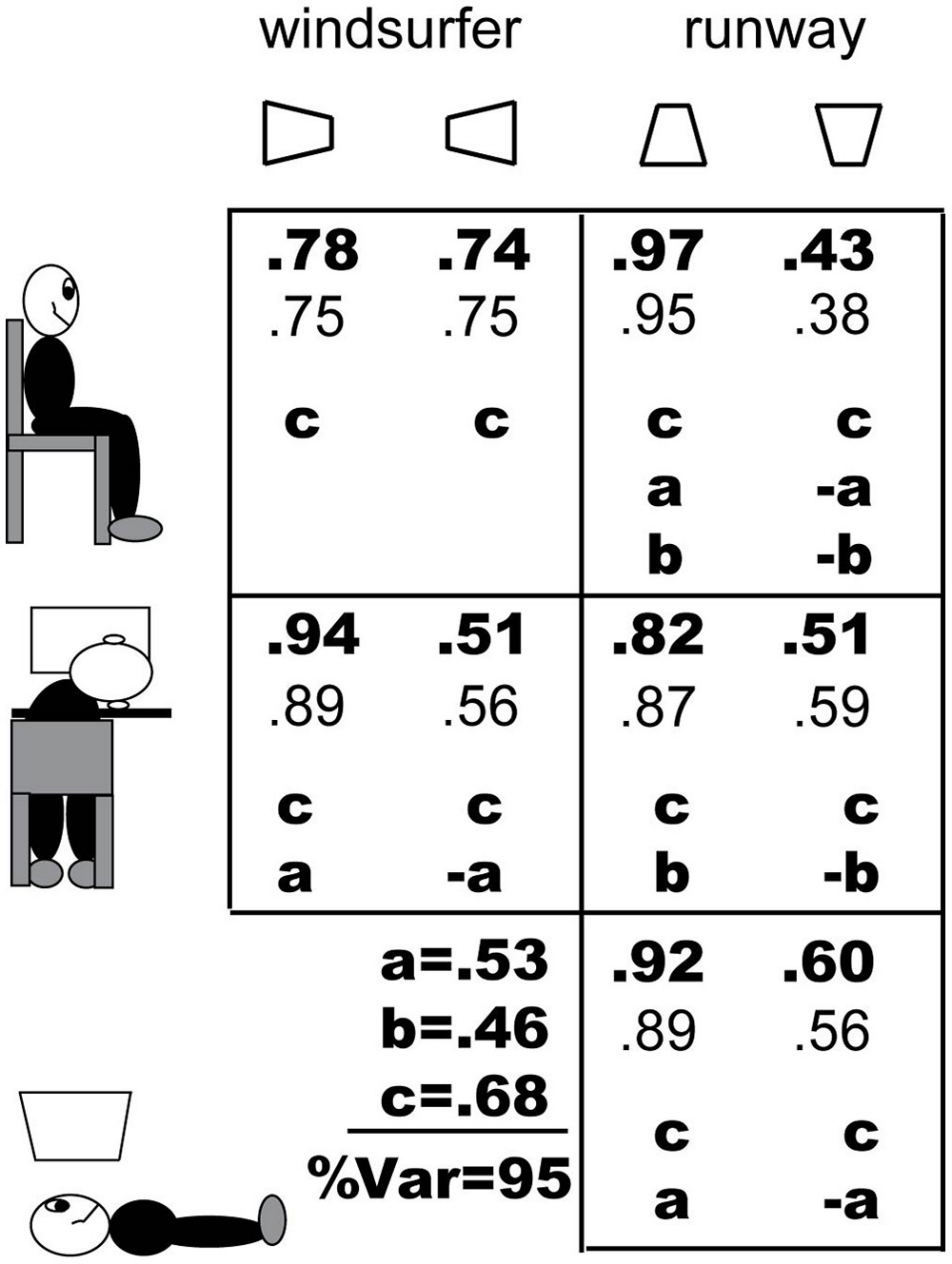
**Proportions of long-in-front responses in the ten experimental conditions, the model predictions, and the three bias factors (*z*-scores) that add to determine perceptual resolution of the ambiguous oscillating trapezoids.** A, long side is lower in a head-centered axis (i.e., in retinal coordinates); b, long side is lower in the world visual field; c, influence of linear perspective interpretation of the trapezoidal configuration on producing a perception of “long side is closer”; %Var is the percent of response variance accounted for by the model.

**Figure 9** shows the viewing conditions, stimuli, results, and model predictions. The viewing conditions are illustrated schematically in the far left column, and the stimuli, indicated as just one of the two identical oscillating trapezoids viewed by the observers are shown in the top row. The 10 data points of the experiments are shown in bold-face type, and immediately below them are the predictions of the model in a lighter weight font. Each panel of **Figure 1** shows which of the three bias factors (see below) of the model, a, b, c, are relevant for that condition and also the sign of the factor. At the bottom left, the estimated values of the bias parameters are given (as inverse Normal coordinates, i.e., *z*-scores).

### Model Computation Summary

**FIGURE 10:**
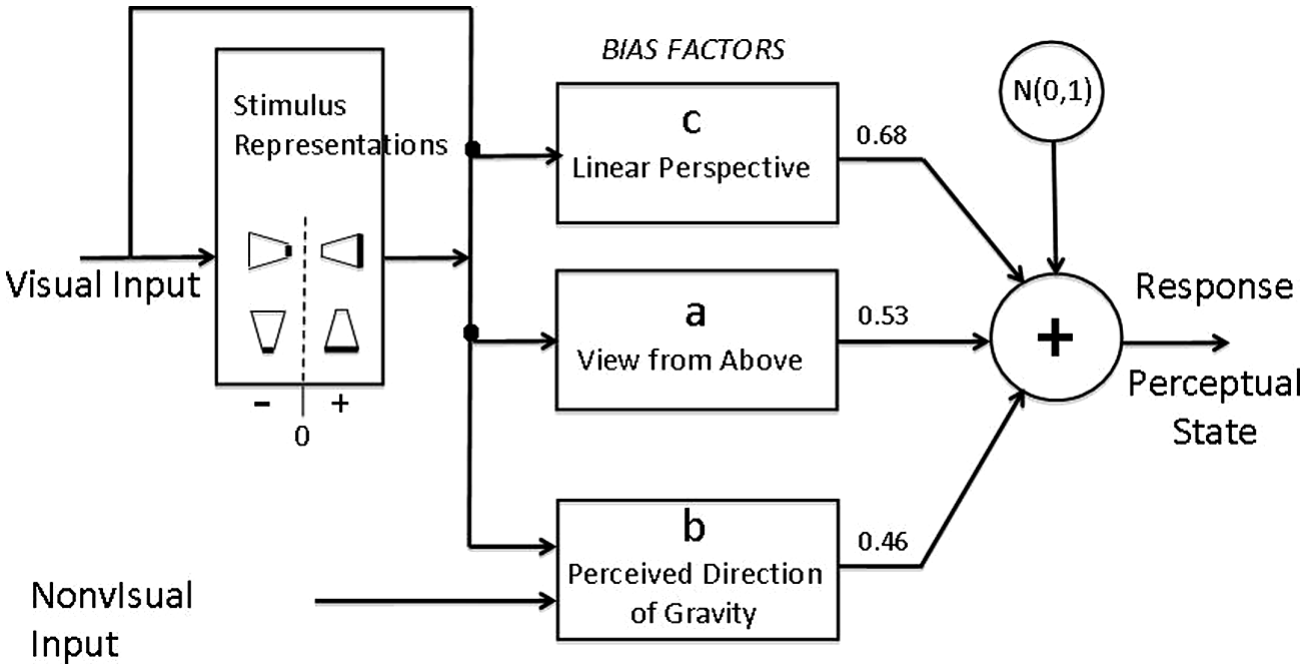
**Block diagram representation of a quantitative model of the bias factors that determine the perceptual resolution of ambiguous oscillating trapezoids, i.e., whether the long side of the trapezoid is perceived to be closer or further from the observer.** Visual input determines the possible stimulus representations (here indicated as trapezoids with the darkened edge as the edge represented as closer) and bias factors. A positive (negative) value for bias factor **(A)** is assigned to stimulus representations in which the long (short) edge has a lower position in head-centered coordinates; a positive (negative) value of **(B)** is assigned when the long (short) edge is lower in the physical world, i.e., in relative to gravity. Bias factor **(C)** is always positive and represents the perspective bias to perceive the long edge as closer than the short edge. *N*(0,1) represents Normally (Gaussian) distributed internal noise with mean zero and variance 1. When the sum of the bias factors and the sampled value of internal noise is positive on a given trial, the output response is a perceptual state (i.e., a particular one of the stimulus representations) in which the long edge is closer to the observer than the short edge, otherwise, the short edge is closer^2^.

The model assumes that there are just two competing perceptual representations: long-side-in-front, short-side-in-front in any of the experimental trapezoidal displays. The relative strength of the long (versus short) side-in-front representation is represented by a real number that is the sum of three situation-determined relevant factors (a,b,c) plus a Normally (Gaussian) distributed random variable that represents internal noise, is added to the relative strength; and if the resulting number is positive, long-side-in front is reported, otherwise, short-side-in front representation that ultimately has the largest strength is one that is reported. The computations of the model are indicated schematically in **Figure 10**.

Models with this basic structure have a long and successful history in psychology (e.g.,Thurstone, 1947; Green and Swets, 1966). In particular, a strength model (based on Thurstone, 1947, Case 5) very successfully describes the combination of factors that determine the perceptual depth interpretation of rotating Necker cubes shown in perspective (Dosher et al., 1986). Both the Dosher et al. (1986) model and the rotating Necker cube displays their model describes are very similar to the present model and the rotating trapezoidal displays dealt with here.

### Factors in the Model

How these bias factors arise from the comparison of two competing representations is illustrated below, as well as an explanation and what they are:

(**a**) Viewing from *above*. Bias factor **a** represents the tendency of the edge that is displayed *lower* in head-centered (i.e., retinal) coordinates to be perceived as closer, i.e., a tendency to perceive objects as being on the ground and viewed from above. When, in retinal coordinates, the long edge of the displayed trapezoid is lower than the short edge, the perceptual representation in which the long edge is in *front* receives a bias increment of **a/2**. The perceptual representation in which the long edge is in *back* receives a bias increment of -**a/2.** Thereby, the **a**-component of the *difference* in bias between the two alternative representations of this display is **a/2 – (**-**a/2) = a**.

When, in retinal coordinates, the long edge of the displayed trapezoid is *higher* than the short edge, the opposite of the previous paragraph occurs. The perceptual representation in which the long edge is in front now receives a bias increment of -**a/2 (**not** a/2** as above). The competing perceptual representation in which the long edge is in back receives a bias increment of **–(**-**a/2) = a/2**. Thereby, the **a**-component of the *difference* in bias between the two alternative representations of this display is -**a/2–a/2 =**-**a**.

Each bias factor represents a particular component of the bias difference in strength between the two alternative perceptual representations. This difference in strength is subject to many sources of noise the net of which is represented as additive internal noise that is Normally distributed with mean 0 and variance 1.

(**b**) *Below* in the world. Factor **b** represents the tendency of the edge that is displayed as being lower in the physical world to be perceived as being closer in 3D space. In these experiments, lower-in-the-physical-world is indistinguishable from lower-in-the-direction-of-gravity. When the long edge is lower in the world, the bias difference between the perceptual representations of long edge in front versus long edge back is **b.** When the long edge is higher in the world, the bias difference between the perceptual representations (long edge in front minus long edge in back) is -**b**.

(**c**) Perspective *configuration*. Perspective configuration **c** refers to the linear-perspective-based trapezoidal illusion—converging stimulus lines represent parallel lines in physical space, the further a point on a line is from the lines’ intersection, the closer it is in physical space. The bias difference between the perceptual representation that is consistent with perspective and the perceptual representation that is inconsistent with perspective is **c**. The bias value of perspective configuration **c** depends only on the shape and movement of the retinal image, independent of its orientation as windsurfer or runway, of head position, or of the relative direction of gravity.

A factor may be positive in one display configuration and either negative or positive in the mirror-image display configuration; the factor is zero in both configurations when it is irrelevant. For example, in Experiment 1, the windsurfer configuration viewed normally, factors **a** and** b** are irrelevant (because the long side is neither up nor down), and factor **c** is positive in both the left and right configuration because both have linear perspective (see **Figure 9**, upper left). Indeed, factor c is positive in all our display configurations because all the configurations are trapezoids and all have a long side. In Experiment 2, the runway configuration viewed normally, factors a and b are both positive when the long side is on the bottom and negative when the long side is on top. The factors in all conditions are shown in **Figure 9**.

The effect of visual context demonstrated in **Figures 2** and **3** was on 3D shape, not on depth orientation, and so was not studied experimentally here and is not represented in the model.

### Model Computations

Let the strength of the long-side-front perceptual representation in condition i, i = 1:10 be s_i_, and the strength of the corresponding short-side-front perceptual representation be s’_i_. The probability of the model choosing the long- versus the short-side-front representation is determined by their difference in strengths Δs_i_ = s_i_–s’_i_.

The basic assumptions of the model is that the strength difference Δs_i_ in condition i is given by a linear combination of the three factors, a,b,c:

(1) $$\Delta s_{i}=\delta_{ia}a+\delta_{ib}b+c+e$$

where the δare either -1,0, or 1 as described above and in **Figure 9**.

The difference in strengths Δs_i_ appropriately transformed is 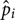 the model’s estimate of the probability advantage of a long-side-front perception over a short-side front perception in condition i. Because of the Normally distributed noise, strength differences are z-scores and are converted to probabilities by a cumulative Normal distribution function with mean = 0 and variance = 1, i.e.,

(2) $$\overset{\wedge }{p}=1/\sqrt{2\pi }\int_{-\infty }^{\Delta s_{i}}c^{\frac{x^{2}}{2}}\mathrm{d}x$$

An optimization procedure based on unconstrained non-linear optimization (Matlab fminsearch) is used to find the values of a,b,c that minimize the summed square difference between the observed p_i_ and predicted 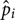. The result of the optimization is the predicted values 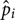 (number below the bold face type) shown in **Figure 9**.

The predicted 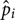 account for 95% of the variance of the data. The root-mean-square difference between observed and predicted probabilities is 0.0439. This is good, not perfect, prediction. But neither are the data perfect. There is great inter-observer variability and there are only 6–8 observers in the various conditions. For example, in the ordinary windsurfer display, based on experience, we expect the difference between the probability of long-side-in-front for left- and for right-pointing trapezoids to be zero. In fact, the difference is 0.78–0.74 = 0.04 – which suggests an upper bound on the accuracy of predictions.

The actual values of a,b,c are of interest. To our knowledge, these are the first quantitative measurements of the strengths of factors that contribute to the perception of ambiguous trapezoids in Ames Window and related displays. The largest factor is perspective configuration, *c* = 0.68. The dominance of perspective configuration (i.e., the 2D represented angle between of lines that are parallel in 3D) is consistent with recent studies that investigate the slant perception of grids. When stationary drawings of skewed grids are viewed from frontal and oblique directions, linear perspective dominates the judgment of perceived slant (Erkelens, 2013, 2015).

However, viewing from above with respect to the head axis, factor *a* = 0.53 is not much smaller than c, indicating a great role for position of the display relative to a head-centered coordinate system. Even factor b (relative position in the world) is 0.46 which also is considerable. The large values of factors a and b explain, in model terms, why viewing a runway configuration with the short side on the bottom causes a reversal of linear perspective and a predominant tendency to see the short side in front: a + b >c, 0.53 + 0.46 = 0.99 > 0.68; being lower both in the retina and in the world trumps being consistent with perspective.

## Summary and Conclusion

(1) We demonstrate an enormously powerful real-world windsurfer illusion that causes the depth orientation of a sail to be completely misinterpreted. The illusion is so powerful, that observers (including even prior reviewers of this article) are completely unaware that they are experiencing an illusion and they cannot reverse it. This is a real-world trapezoidal illusion.
(2) Illustrations of static trapezoidal shapes painted on walls, ceilings, and floors of rooms shows that the perceived shape of identical 2D trapezoids can be heavily influenced by this perspective context but perceived 3D depth orientation is less influenced.
(3) When viewing dynamic oscillating trapezoids (Experiment 1) in a windsurfer configuration, observers perceive the longer side in front 74% of the time indicating a strong trapezoid effect based on implicit perspective (similar to the Ames window). The location of the axis around which the trapezoid rotates is a smaller factor in determining perceived depth than the other factors considered here. The longer sides of left- and of right-pointing trapezoids are statistically equally likely to be perceived in front.
(4) When the displays of Experiment 1 were rotated 90° to the right, the left-pointing windsurfers becomes normal runways and the right-pointing windsurfers become upside down runways. The previous left–right symmetry is broken; the long, bottom side of the normal runway configuration is perceived in front 97%. For upside-down runways, with the long side on top, it is perceived in front only 43%, thereby overcoming the trapezoid effect in which the long side is perceived in front more than 50%. A surprisingly large and consistent change in depth percept arises with what may seem at first glance to be a very small change in visual stimulation: a trapezoidal figure is rotated 90°. This pattern is consistent with a strong bias to interpret scenes as being viewed from above in a head-centered frame of reference if possible (here, possible in all experiments except Experiment 1).
(5) In Experiment 3, the displays of Experiments 1 and 2 were viewed with the head tilted 90° to the right. When the retina is presented a symmetric windsurfer configuration but the real world contains a runway configuration, the asymmetric pattern of runway perceptions is observed, as it is when retina is presented runways and the real-world contains windsurfer configuration. This pattern of results was consistent with an implicit tendency to interpret patterns as being viewed from above.
(6) Experiment 4 tested whether viewing from above was related to the direction of gravity. Observers lay on the ground and viewed runway configuration stimuli projected on the ceiling. Perceptions were similar to Experiment 3 in which the head was tilted to also produce a runway configuration on the retina while viewing a real-world windsurfer configuration.
(7) A model was proposed in which three independent bias factors (**a,b,c**) derived from the display configuration add linearly to determine the probability of perceiving the long trapezoidal side as being “in front” in the ten experimental conditions. Factor **a** assumes viewing from above with respect to a head-centered (retinal) frame of reference and thereby bias the lower trapezoidal edge in a runway configuration to be perceived as closer to the observer than the upper edge; factor b represents the bias to perceive the trapezoidal edge that is lower in the physical world as being closer, factor c represents a perspective bias to perceive the long edge of a rotating trapezoid as closer independent of the trapezoid’s orientation on the retina and of any of the other factors. Internal Gaussian distributed noise (mean zero, variance 1) determines trial-to-trial variability. The values of the factors, which indicate their relative importance are: *a* = 0.65, *b* = 0.41, *c* = 0.73. The model accounts for 93% of the variance of the data. We conclude that a simple computational model gives a reasonable account of the influence of three significant factors–linear perspective, retinal position, world position that determine the perception of ambiguous rotating trapezoids.

## Conflict of Interest Statement

The authors declare that the research was conducted in the absence of any commercial or financial relationships that could be construed as a potential conflict of interest.

## Footnotes

2 In an actual brain model (versus the computational model utilized here) the bias factors would interact with (feed back to) the stimulus representations to determine the winning perceptual state, such complications are not relevant here.
